# Genome assemblies of the liverwort *Blasia pusilla* uncover a well-defined pseudoautosomal region on homomorphic UV sex chromosomes

**DOI:** 10.1186/s13059-026-04101-2

**Published:** 2026-05-18

**Authors:** Yuling Yue, Gaurav Sablok, Xiaolan He, Jaakko Hyvönen, Shanshan Dong, Giacomo Potente, Yang Liu, Péter Szövényi

**Affiliations:** 1 Department of Systematic and Evolutionary Botany, University of Zurich, Zollikerstrasse 107, Zurich, 8008 Switzerland; 2 Zurich-Basel Plant Science Center, Zurich, Switzerland; 3 Botany and Mycology Unit, Finnish Museum of Natural History, University of Helsinki, PO Box 7, Helsinki, FIN-00014 Finland; 4 Key Laboratory of Southern Subtropical Plant Diversity, Fairy Lake Botanical Garden & Chinese Academy of Sciences, Shenzhen, China; 5 BGI, BGI Research, Wuhan, China

## Abstract

**Background:**

Sex chromosomes evolve from autosomes and are expected to be initially homomorphic, though they may become heteromorphic over time. In diploid systems, one sex chromosome often degenerates, whereas homomorphy should persist longer in organisms with haploid UV sex chromosomes. Intriguingly, both homomorphic and heteromorphic UV sex chromosomes are prevalent in liverworts, yet the genomic structure and evolutionary origins of homomorphic sex chromosomes remain unexplored.

**Results:**

We investigate the structure and gene content of the homomorphic UV sex chromosomes of the liverwort *Blasia pusilla*. We uncover a previously undescribed organization comprising a large recombining pseudoautosomal region (PAR) and a terminal, non-recombining sex-determining region (SDR) on both U and V chromosomes. Notably, the SDR of *Blasia pusilla* is homologous to the compact SDR of the heteromorphic UV chromosomes in *Marchantia polymorpha*, a liverwort species lacking well-defined PARs.

**Conclusions:**

Our results show that the UV sex chromosomes of *Blasia pusilla* have a well-defined SDR and a PAR. We hypothesize that this organization is more prevalent in the homomorphic UV sex chromosomes of liverworts than previously expected and could potentially represent an ancestral feature of the Marchantiidae subclass. However, we cannot exclude the possibility of an origin via fusion between an autosome and a micro-sex chromosome, similar to the UV chromosomes of *Marchantia polymorpha*. Our study has significant implications for understanding sex chromosome evolution in plants possessing a UV sex-determining system.

**Supplementary Information:**

The online version contains supplementary material available at 10.1186/s13059-026-04101-2.

## Background

Sex chromosomes are thought to evolve from a pair of ordinary autosomes through the emergence of an initial sex determining-region (SDR) possessing sex-determining genes [1]. Therefore, young sex chromosomes are expected to be homomorphic (e.g., similar in size and cytological appearance) except for the SDR that is divergent and often protected from recombination [2]. Over time, recombination suppression may expand beyond the SDR, leading to the extensive divergence and potential heteromorphy (unequal size or structure) of sex chromosomes [3]. In diploid systems where sex is determined by a pair of chromosomes (X/Y and Z/W), heteromorphy is thought to arise due to the asymmetry in recombination suppression between sex chromosomes (X and Z recombine whereas Y and W do not) leading to the preferential degeneration of the non-recombining sex chromosome [4]. By contrast, when sex is determined by a single female (U) or male (V) chromosome in a haploid system, recombination suppression is symmetric and both U and V chromosomes should undergo similar levels of degeneration, potentially allowing them to remain microscopically homomorphic over extended evolutionary timescales [5, 6].

**Fig. 1:**
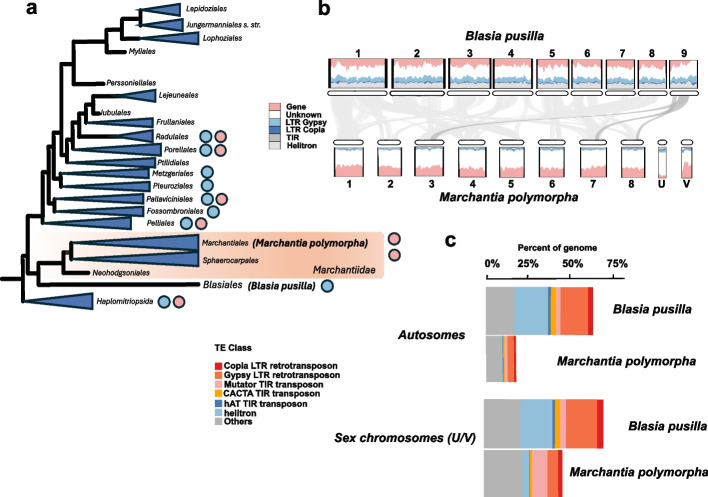
Occurrence of homo- and heteromorphic sex chromosomes in liverworts and comparison of the *Blasia pusilla* and the *Marchantia polymorpha* genomes. **a** A simplified phylogenetic tree of liverworts [22]. Showing the position of *B. pusilla* and the subclass of Marchantiidae liverworts (highlighted with a light red rectangle). Blue circles refer to homomorphic whereas pink circles to heteromorphic sex chromosomes following the information provided in Allen C [7]. **b** Chromosome-scale synteny between the *B. pusilla* male and the *M. polymorpha* genomes. Collinear blocks are connected by grey ribbons. Collinear blocks on chormosome nine of *B. pusilla* are in darker color. Repeat and gene density along the chromosomes are also shown. Chromosomes are drawn to scale. **c** Overall abundance of repeats across the *B. pusilla* male and *M. polymorpha* genomes

In several photosynthetic eukaryotic lineages, such as green, red, and brown algae, as well as bryophytes, sex is determined by UV chromosomes [7–10]. Consistent with the theoretical expectation of symmetric divergence, most sex chromosomes investigated in red and brown algae appear to be ancestrally homomorphic and remain so for many hundred million years [11, 12]. Yet some taxonomic groups possess a surprisingly large number of species with highly heteromorphic sex chromosomes. For instance, liverworts, whose dioicous condition and UV system are at least 430 Myr old, harbor both homomorphic and heteromorphic sex chromosomes which occur in a scattered pattern across the phylogeny (Fig. 1a) [7, 13–15]. When sex chromosomes are heteromorphic, the U is often considerably larger than the V, and both are significantly smaller than the autosomes, forming so-called microchromosomes, such as the sex chromosomes of the liverwort model species *Marchantia polymorpha* [15–17]. The genomic sequences and gene content of heteromorphic UV sex chromosomes are well-characterized, and their functional significance in sexual reproduction has intensively been investigated in *M. polymorpha* [15, 18]. By contrast, homomorphic sex chromosomes have only been described through cytological observations, and their genome sequence, structure, and gene content remain to be determined (Fig. 1a) [7, 13, 19, 20]. The complete lack of information on the genomes of homomorphic liverwort sex chromosomes prevents any conclusions to be drawn about their evolutionary relationship with their heteromorphic counterparts, including their ancestral or derived nature.

To address this gap, we sequenced the genomes of a male and a female isolate of *Blasia pusilla* L., a liverwort possessing cytologically homomorphic UV sex chromosomes [7, 19] (Fig. 1a). We found that both *B. pusilla* sex chromosomes comprise a pseudoautosomal region (PAR) and a shorter, terminally located, non-recombining sex-determining region (SDR), a structure which has not been observed in liverworts before. This study therefore provides the first chromosome-level genomic characterization of homomorphic UV sex chromosomes in liverworts. This is also the first time that a well-defined PAR is discovered on the sex chromosomes of a liverwort. Based on these results and considering the phylogenetic position of *B. pusilla*, we propose that homomorphy, as well as the PAR-SDR structure, may represent an ancestral condition within the Marchantiidae subclass [21, 22]. Furthermore, this implies that heteromorphic sex chromosomes evolved through the reduction or complete loss of the PAR. However, in the absence of high-quality genomic data from other liverworts with homomorphic sex chromosomes, it cannot be entirely ruled out that the homomorphic sex chromosomes of *B. pusilla* originated through fusion of an autosome and a micro-sex chromosome. Further sampling of liverwort karyotypes and genomes will be required to distinguish between these alternatives.

## Results

### The *Blasia pusilla* genome does not possess microchromosome-like sex chromosomes typical for dioicous liverworts investigated so far

We assembled the genomes of an axenic male (Finnish strain) and a female isolate (Chinese strain) of *B. pusilla* using long reads (PacBio and ONT). Final assembly sizes were 377 Mbp and 390 Mbp, respectively (Additional file 1: Tables S1 and S2), consistent with their k-mer based genome size estimates (Additional file 1: Table S3 and Additional file 2: Fig. S1), suggesting that the assemblies are nearly complete. After Hi-C scaffolding, the male assembly consisted of nine chromosome-sized scaffolds, in accordance with previous cytological observations [19] (Additional file 2: Figs. S2 and S3). The female assembly was less contiguous and contained 14 final scaffolds after Hi-C and reference-guided scaffolding against the male assembly (Additional file 2: Figs. S2 and S3). Telomeres were more completely assembled in the female than in the male assembly (Additional file 2: Fig. S4). Unlike other Marchantiidae genomes that have been sequenced so far, the *B. pusilla* genomes comprised three larger and six smaller chromosomes, none of which are microchromosome-like sex chromosomes described in other dioicous liverworts [17, 23–25] (Fig. 1b). The two *B. pusilla* genomes contained slightly fewer genes (~ 17, 000 in the Finnish, ~ 18, 000 in the Chinese strain) than *M. polymorpha* (~ 19, 000) [16] but nearly twice as many repetitive elements (~ 61% vs. 32%), accounting for most of the 100 Mbp difference in genome size between the two species (Fig. 1c and Additional file 1: Table S5). Transposable elements (TEs) covered 54.57% of the *B. pusilla* male genome, with Helitrons unusually abundant (~ 16.81% of all annotated TEs, Fig. 1c and Additional file 1: Table S5). Like other liverworts, the *B. pusilla* genome showed no evidence of whole genome duplication (Additional file 2: Fig. S5). BUSCO scores implied that the gene space of both assemblies was well-covered (Additional file 1: Table S4).

### Sex chromosomes of *Blasia pusilla* consist of a pseudoautosomal (PAR) and a terminal sex-determining region (SDR)

To identify the sex chromosomes in *B. pusilla*, we first carried out collinearity analyses with the *M. polymorpha* genome. In contrast to earlier reports on Marchantiidae liverworts [24–28], we did not detect one-to-one synteny between *B. pusilla* and *M. polymorpha* chromosomes (Fig. 1b). Instead, each *B. pusilla* chromosome showed synteny with two or more *M. polymorpha* chromosomes, indicating multiple chromosomal fissions, fusions, and translocations since the divergence of the two species (Fig. 1b). Furthermore, we did not find any collinear blocks between the U/V chromosomes of *M. polymorpha* and *B. pusilla* (Fig. 1b) [15, 24, 28]. One exception was chromosome nine, which contained a short terminal segment that lacked collinearity with any of the *M. polymorpha* chromosomes, making it a candidate sex chromosome (Fig. 1b).

**Fig. 2:**
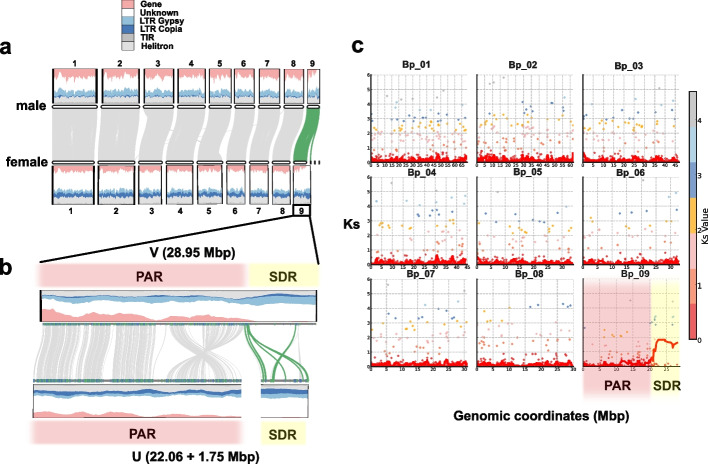
Sex chromosome structure in *Blasia pusilla*. **a** Comparison of the male and female *B. pusilla* genomes. Gene and transposable element (TE) density is shown above the chromosomes, collinear regions among autosomes (Bp_01—08) are connected with gray ribbons. Collinear blocks of chromosome nine are shown in green color. **b** Comparison of chromosome nine between the male and female *B. pusilla* assemblies. Gene and transposable element (TE) densities are shown above the chromosome. In the pseudoautosomal region (PAR), collinear blocks are depicted as gray ribbons whereas in the sex determining region (SDR) gene pairs homologous to *M. polymorpha* gametologs are connected with green ribbons. The PAR and the SDR in the female assembly lie on two separate scaffolds. **c** Number of synonymous substitutions per synonymous sites (K_s_) between homologous gene pairs of the male and female assemblies. Each dot represents an individual male–female gene pair, and the red line refers to the average K_s_ in a 1 Mbp sliding window. While most chromosomes show uniformly low divergence, chromosome nine exhibits a marked increase in K_s_ values in its terminal region

To investigate whether chromosome nine represented the sex chromosome, we compared its genomic features with those of the other chromosomes, expecting sex chromosomes to show elevated transposable element (TE) content, reduced gene density, and extensive structural rearrangements compared to autosomes [15–17, 26–28]. In the more contiguous male *B. pusilla* genome, chromosome nine showed distinct structural features that set it apart from the other eight chromosomes (Figs. 1b, 2a, b). Approximately two-thirds of chromosome nine (0—21.41 Mbp) exhibited gene and repeat density similar to chromosomes 1—8; we therefore refer to this autosome-like part as the pseudoautosomal region (PAR). Although the PAR was similar to the autosomes, it differed significantly in some respects. In particular, the PAR showed somewhat reduced gene density, and its genes were slightly shorter, had fewer exons, and lower GC/GC3 content than those of the autosomes (Additional file 2: Fig. S6). Furthermore, it was also slightly enriched for orphan genes (genes without significant homology to a large set of liverwort proteomes) compared to the autosomes (Additional file 2: Fig. S7). In contrast to the PAR, the terminal one-third of chromosome nine (21.41—30 Mbp) showed markedly lower gene density (42 genes/8.59 Mbp = 4.90 gene/Mbp) and a substantially elevated abundance of Copia and Gypsy long terminal repeat (LTR) retrotransposons, often arranged in tandem, and was therefore designated as the sex-determining region (SDR) (Fig. 2a and Additional file 2: Figs. S6 and S8). While we were able to obtain a contiguous assembly for chromosome nine in the male *B. pusilla* strain, in the female assembly the SDR and the PAR were located on two separate scaffolds probably because of the vast number of tandem repeats of the SDR. Nevertheless, the repeat content and gene density of the female SDR were similar to those of the male SDR (Fig. 2b). Furthermore, most genes of the female SDR had a homolog in the male SDR, suggesting that only a small proportion of the female SDR may be missing (Additional file 1: Tables S7 and S8). We further found that the U and V PARs showed high sequence similarity, with low synonymous divergence (K_s_) between male and female allelic pairs as well as extensive synteny, whereas the SDRs possessed considerably higher structural, gene order, and silent site (elevated K_s_) divergence (Fig. 2b, c).

### The SDR possesses ancestral gametologs as well as recently captured genes and shows suppressed recombination

**Fig. 3:**
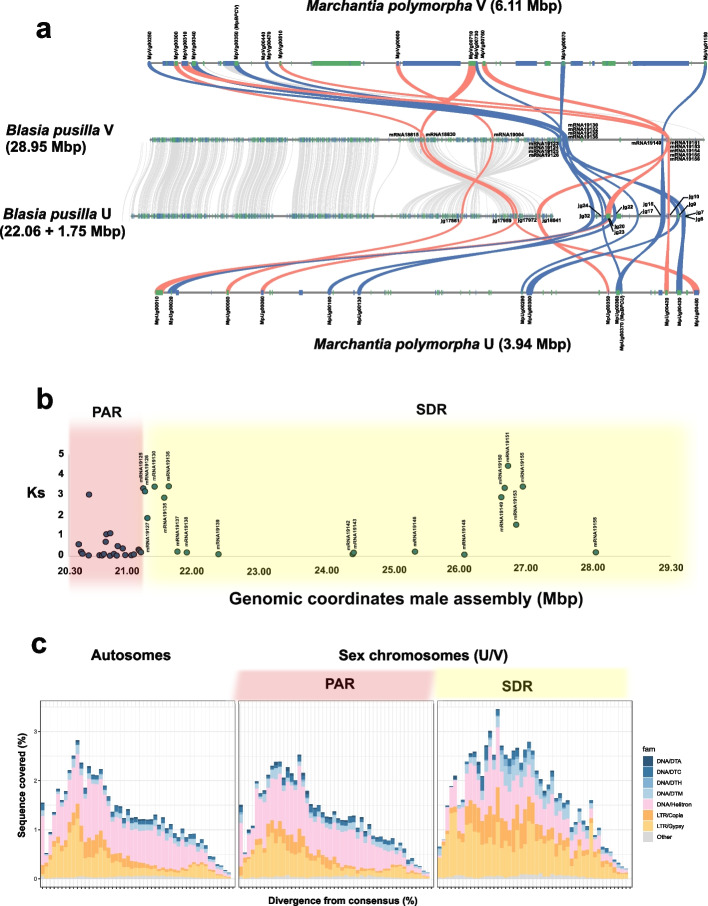
Homology, evolutionary strata and repeat landscapes of the pseudoautosomal (PAR) and sex-determining regions (SDR) of *Blasia pusilla* chromosome nine. **a** Homology of *B. pusilla* chromosome nine genes with *M. polymorpha* UV gametologs. Blue and green boxes indicate the positions of gene models on the forward and reverse strands, respectively. Grey ribbons connect homologous genes of the male and female *B. pusilla* assemblies. *B. pusilla* homologs of *M. polymorpha* UV gametologs are connected with colored ribbons. Homologs whose divergence predates the split of *B. pusilla* and *M. polymorpha* are depicted in blue whereas those postdating it in red. Only UV gametologs are shown on the *M. polymorpha* chromosomes. **b** K_s_ values between male and female gene pairs in the SDR and in the adjacent region of the PAR in *B. pusilla.* **c** Repeat landscapes for the *B. pusilla* autosomes, the PAR and the SDR

To further test the sex chromosome identity of chromosome nine, we searched for homologs of *M. polymorpha* UV gametologs (i.e. homologous genes on the U and V sex chromosomes derived from the same ancestral locus) in the *B. pusilla* genome, which we expected to be localized in the SDR of *B. pusilla*. To do so, we constructed gene trees using *M. polymorpha* gametologs and their *B. pusilla* homologs, as well as homologs from other liverwort species (see “ Methods” and Additional file 1: Table S6). To compensate for potential incompleteness of the female SDR assembly, we also included transcriptomic data from two additional female accessions of *B. pusilla* (see Additional file 1: Table S7). We found that the male SDR of *B. pusilla* contained 11 homologs of the 19 *M. polymorpha* gametolog pairs, including the homolog of the *BPCV* gene known to be essential for sex determination in *M. polymorpha* [15, 18], with a further four homologs occurring in the PAR (Fig. 3a, Additional file 1: Table S7).

Next, we investigated whether gametolog phylogenies supported recombination suppression in the SDR, an expected feature of many SDRs ensuring sex-linked segregation [1] (see “ Methods” and Additional file 2: Fig. S9). Indeed, we found that seven *B. pusilla* homologs of *M. polymorpha* gametologs located mainly adjacent to the junction of the SDR and the PAR on the V chromosome predated the divergence of these two species and formed distinct male and female allelic clades alongside their *Marchantia* counterparts, as would be expected under recombination suppression (Fig. 3a, Additional file 2: Fig. S9). The deeply rooted origin of these gametologs was also confirmed by the high K_s_ values between their U and V alleles (Figs. 2c, 3b, Additional file 1: Table S10). Six of these seven *B. pusilla* gametolog pairs were homologous to *M. polymorpha* gametologs previously identified as being part of the ancestral non-recombining stratum of liverwort sex chromosomes (*MpVg00340*, *MpVg00350*, *MpVg00440*, *MpVg00470*, *MpVg00970*, *MpVg01150*, see oldest stratum in [15]) (Fig. 1a). The remaining ancestral *M. polymorpha* gametolog, *MpVg00980*, had a *B. pusilla *homolog showing autosomal localization (Additional file 1: Table S7). By contrast, most *B. pusilla* homologs of *M. polymorpha* gametologs found in the PAR, or close to the telomere of the SDR (see the homolog of *MpVg01150* as an exception), showed divergence postdating the split between the two species (Fig. 3a, Additional file 1: Tables S7 and S10, Additional file 2: Fig. S9,). In addition to these shared gametologs, the male and female SDRs of *B. pusilla* each contained 18 and 25 genes, respectively, that had either no homologs in *M. polymorpha* or the homologs were found on *M. polymorpha* autosomes (Fig. 3a, Additional file 1: Tables S7 and S8).

We further asked whether the SDR captured genes in multiple waves, and whether older and younger genes are spatially clustered in the SDR, as they do in species, showing evidence of evolutionary strata [1]. We observed that gametolog K_s_ values in the *B. pusilla* SDR formed two well-defined K_s_ classes, corresponding to older and younger divergence levels (Fig. 3b). Furthermore, we found that gametolog pairs with greater and smaller K_s_ values occurred intermixed throughout the male as well as the female SDR in *B. pusilla* and showed no increasing or decreasing trend from a focal point (Fig. 3a, b) consistent with previous observations for bryophyte UV chromosomes [15, 17, 33–37]. Interestingly, the part of the PAR directly adjacent to the SDR appeared to carry an inversion between the U and V chromosomes and contained three *B. pusilla* homologs of *M. polymorpha* gametologs whose age postdates the split of the two species (Fig. 3a). Repeat landscape analysis showed that the SDR contained a higher proportion of older TEs than the PAR (Fig. 3c and Additional file 2: Fig. S10), suggesting that evolutionary dynamics of TEs in the SDR and the PAR indeed differ.

### The BPCU/V-SUF-FGMYB regulatory module is present in *B. pusilla*

**Fig. 4:**
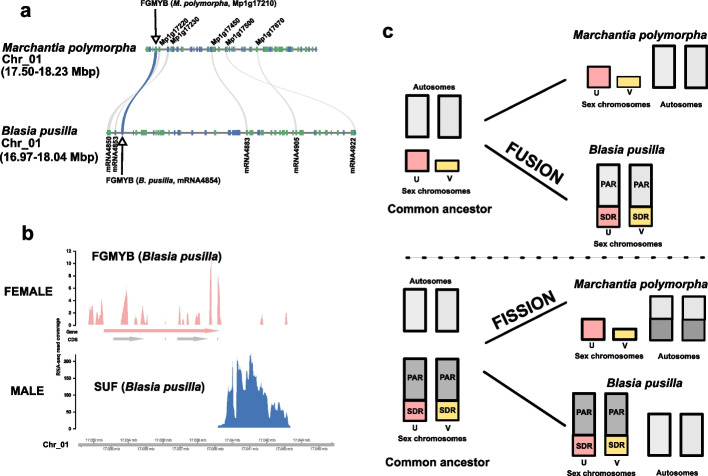
Sex determination and the putative evolutionary origin of homomorphic sex chromosomes in *Blasia pusilla*. **a** Genomic synteny between *B. pusilla* and *M. polymorpha* around the *FGMYB* ortholog of *B. pusilla.* The grey lines connect homologous genes. The blue ribbon highlights the orthologous *FGMYB* gene pairs (*Mp017210* in *M. polymorpha*, *mRNA 4754* in *B. pusilla). ***b** RNA-seq read coverage of the *B. pusilla FGMYB* ortholog and a putative SUF antisense RNA in female (red, top panel) and male isolates (blue, bottom panel). Gene model annotations are shown at the top. **c** Two mutually exclusive models potentially explaining the evolutionary origin of homomorphic sex chromosomes in *B. pusilla*. Sex chromosomes are shown in red (U) and yellow (V) whereas autosomes are in gray color. PAR: pseudoautosomal region, SDR: sex-determining region

In *M. polymorpha,* the sex-linked transcription factors BPCU and BPCV, together with the autosomal long non-coding RNA SUF and the autosomal transcription factor FGMYB, form the core regulatory module controlling reproductive organ development and sex-determination [17, 18, 39]*.* Given that this regulatory network is thought to be deeply conserved in liverworts [15, 25], we therefore investigated the presence, genomic location, and expression patterns of these four core sex-determining genes in *B. pusilla*. We found orthologs of *BPCU* and *BPCV* residing within the U and V SDRs, respectively, whereas a single ortholog of *FGMYB* was present on chromosome one. Microsynteny of the genomic sequence flanking the *FGMYB* gene is conserved between *B. pusilla* and *M. polymorpha* [15, 16] (Fig. 4a). Transcriptomes from gametophytes revealed low expression of *FGMYB* in females, and no detectable expression in males (Fig. 4b). In contrast, an antisense *SUF* transcript was observed in males but not in females, consistent with the expression pattern described in *M. polymorpha* [15, 18, 29].

## Discussion

### The *B. pusilla* sex chromosome is homomorphic with a clear PAR-SDR architecture

The most surprising finding of our study is the discovery of a well-defined PAR and SDR constituting the UV sex chromosomes in *B. pusilla*. To our knowledge, this is the first genome-scale finding of a homomorphic liverwort sex chromosome with a clearly defined PAR-SDR architecture. This is in contrast with previously characterized bryophyte (mosses, liverworts and hornworts) UV chromosomes, in which gene and repeat density are more uniformly distributed across the entire sex chromosome, and a distinct PAR has not been identified [16, 28, 30–34]. In addition, our study confirmed previous cytological observations that the U and V chromosomes of *B. pusilla* are not heteromorphic microchromosomes, i.e. they do not significantly differ in appearance and are comparable in size to autosomes [7, 19]. In light of these observations, two questions arise: is the presence of UV chromosomes with a well-defined PAR and SDR unique to *B. pusilla*, or does it occur in other liverworts? And do liverwort homomorphic sex chromosomes tend to have this structure? Without further data, it is difficult to answer these questions, but we speculate that the lack of data on liverwort sex chromosomes with a PAR-SDR structure is probably the result of sampling bias. This is because previous studies investigating bryophyte sex chromosomes have focused primarily on species with highly heteromorphic U/V chromosomes, as these are more easily studied using cytogenetics methods [16, 28, 30–34]. Therefore, we assume that liverwort sex chromosomes with a PAR-SDR structure may be more common than previously thought, potentially representing a frequent feature of homomorphic sex chromosomes. Nevertheless, this assumption must be rigorously tested in the future by obtaining high-quality genome assemblies from additional liverworts with both homomorphic and heteromorphic sex chromosomes.

### The *B. pusilla* SDR houses a dynamic gene set and is homologous to the UV sex chromosomes of *M. polymorpha*

Our data show that chromosome nine of *B. pusilla* carries a terminally located SDR with suppressed recombination occupying approximately 1/3rd of the chromosome. Furthermore, comparative analysis of genes between the UV SDR of *B. pusilla* and the UV micro-sex chromosomes of *M. polymorpha* implies that they are homologous and have shared ancestry. More specifically, we found that six out of seven *B. pusilla* homologs of *M. polymorpha* gametologs were localized to the *B. pusilla* SDR indicating that these gametologs, as well as the genomic segments carrying them, were already present in the common ancestor of *M. polymorpha* and *B. pusilla* (Fig. 1a). Phylogenetic analysis of these gametologs also implies that they reside in genomic regions where recombination was already suppressed in the two species' common ancestor. By contrast, the *B. pusilla* homolog of one *M. polymorpha* UV gametolog did not occur on chromosome nine but was found on a *B. pusilla* autosome, implying that this gene experienced autosomal translocation in *B. pusilla* given that previous analysis on a vast number of liverwort species indicated [15] that it was part of the non-recombining gene set shared by all extant liverworts (see Fig. 1a). Besides the shared gametologs, the *B. pusilla* SDR also possessed a considerable number of additional gametologs as well as U- and V-specific genes which either had no homologs in *M. polymorpha* or the homologs were found on *M. polymorph*a autosomes (Additional file 1: Table S8). Many of these genes have paralogs elsewhere in the *B. pusilla* genome, consistent with a scenario involving gene duplication and transposition to the UV chromosomes. Therefore, these genes represent independent and recent gene captures by the U and V SDRs in *B. pusilla*.

Collectively, these lines of evidence suggest that the SDR contains a mixture of deeply conserved (predating the split of all extant liverworts) and recently acquired gametologs as well as UV-specific genes. Therefore, the core set of gametologs is embedded in a dynamic set of genes gained or lost by the SDR in a lineage-specific manner. Our data also confirms previous findings that some parts of the SDR carrying the core set of gametologs were already non-recombining in the common ancestor of all extant liverworts (Fig. 3a and Additional file 1: Tables S7 and S8) [15].

### The *B. pusilla* PAR and SDR show divergent evolutionary trajectories

In all bryophyte species investigated so far, UV sex chromosomes have been found to be enriched for TEs, have lower gene density, and show more extensive gene order rearrangements compared to the autosomes [16, 28, 30–34]. We found that the *B. pusilla* SDR showed similar patterns that are typical of genomic regions with suppressed recombination [1, 35, 36]: it was enriched for TEs, showed decreased gene density, extensive rearrangements, and stronger divergence between U and V haplotypes compared to the PAR and the autosomes. The *B. pusilla* SDR also contained a higher proportion of older TEs (Fig. 3c and Additional file 2: Fig. S10) than the PAR and the autosomes. This suggests less effective purging of TEs in the SDR due to suppressed recombination. Alternatively, the greater abundance of TEs in the SDR could be associated with, or facilitated by, the establishment of suppressed recombination throughout the SDR.

By contrasts, approximately 2/3rd of chromosome nine of *B. pusilla* was occupied by the pseudoautosomal region (PAR). We refer to this region as the PAR because it markedly differed from the SDR and resembled the autosomes in multiple respects. In particular, the PAR's gene and TE density were very similar to those of the autosomes, as opposed to the SDR. Furthermore, the lower K_s_ divergence of the male and female alleles of the PAR genes suggested extensive recombination, in contrast to the SDR, that showed elevated K_s_ values and suppressed recombination. Both theoretical models and experimental evidence suggest that plant and animal PARs are similar to autosomes but differ from them in multiple respects due to their sex-linkage [37–39]. In line with this, we found that the PAR of *B. pusilla* exhibited some genomic features that were different from those of the autosomes, and that these features were shared with the PAR of the brown alga *Ectocarpus siliculosus* [38]. In particular, it was similarly enriched for young genes that were shorter and had fewer as well as shorter exons than their autosomal counterparts (Additional file 2: Figs. S6 and S7). This suggests that the evolutionary trajectories of brown algal and liverwort PARs could have been similar. Collectively, our data show that genomic characteristics (gene and repeat density and age distribution) markedly differ between the PAR and the SDR implying their distinct evolutionary trajectories. The presence of an SDR containing deeply shared gametologs suggests that its genomic characteristics as well as evolutionary trajectory were established very early on, prior to the split of *B. pusilla* and *M. polymorpha* [15, 24].

### UV sex chromosome structure of *B. pusilla* resembles those of green, red and brown algae

It is important to note that the PAR-SDR structure and homomorphy of *B. pusilla* sex chromosomes resemble those of red and brown algal UV chromosomes [11, 12, 40]. Furthermore, the SDRs of brown and red algae, share multiple features with that of *B. pusilla,* including low gene density, extensively rearranged gene order, elevated TE abundance, a lack of recombination, and highly divergent male and female alleles. *B. pusilla* U/V chromosomes are also similar to green algal mating-type (MT) chromosomes to some extent, in which an internal mating-type region (analogous to the SDR) is surrounded by terminal autosomal segments (analogous to the PAR) [41–43]. Nevertheless, green algal MT chromosomes are more diverse than UV chromosomes of red and brown algae. For instance, in some species MT regions possess relatively young genes and are not significantly enriched for TEs compared to other chromosomes. Despite these differences, MT and UV chromosomes of green, brown and red algae appear to be mainly homomorphic, e.g. similar in size and cytological appearance, and show only slight signs of degeneration [43], consistent with the assertion that homomorphy and the presence of PARs and SDRs are generally ancestral features of UV sex chromosomes which can persists for a long time [1, 43].

By contrast, it is estimated that only about half of liverwort species possess homomorphic sex chromosomes [7, 13]. Although theory predicts the long-term persistence of sex chromosome homomorphy in UV systems, the evolutionary forces that maintain an extensive and well-defined PAR in brown, red and green algae are poorly understood [6, 38, 44]. Assuming that most homomorphic sex chromosomes of liverworts possess a well-defined PAR, similar to that of *B. pusilla,* while highly heteromorphic sex chromosomes mainly consist of an SDR, suggests that liverwort PARs may be more dynamic than those of green, red and brown algae. The fact that liverworts with highly reduced or undetectable PARs can properly pair and segregate during meiosis suggests that most of the PAR can be lost without significant fitness costs. This is similar to the X and Y chromosomes in humans, in which only a very short (2.7 Mbp (PAR1)) segment of the sex chromosomes is sufficient to ensure proper pairing and segregation [45]. We currently have no adequate explanation as to why an extensive PAR would be maintained in all the investigated brown and red algal sex chromosomes and green algal mating-type (MT) chromosomes, yet not in liverworts. Further research is needed to determine whether this difference is linked to divergent aspects of their general biology, including physiology and ecology.

### Is the PAR-SDR structure ancestral to Marchantiidae liverworts?

The discovery of a PAR-SDR structure in *B. pusilla* raises questions about its evolutionary origins, prompting us to propose two alternative hypotheses that are supported by our data. (i) It is possible that homomorphic sex chromosomes with a well-defined PAR and SDR with suppressed recombination represent the ancestral condition in Marchantiidae liverworts (Figs. 1a and 4c). In this scenario, the heteromorphic micro-sex chromosomes found in *M. polymorpha* may have evolved via reduction as well as translocation of the PAR to the autosomes (Fig. 4c, FISSION). The translocation of the PAR may have occurred via immediate chromosomal rearrangements, such as those that occur during chromotripsis [46], or more gradually, involving the expansion of the SDR into the PAR. The observation that the *B. pusilla* PAR is syntenic with several *M. polymorpha* autosomes is compatible with transfer of the PAR to autosomes in one or multiple steps (Fig. 1b). Furthermore, we observed a large inversion between the U and V sex chromosomes of *B. pusilla* located at the junction of the PAR and the SDR. Therefore, we speculate that such inversions could have contributed to the expansion of the SDR and the gradual reduction of the PAR leading to the evolution of highly heteromorphic microchromosomes such as the UV sex chromosomes of the model liverwort *M. polymorpha* [15, 16]*.* Intriguingly, this inverted region contains three *B. pusilla* homologs of the *M. polymorpha* gametologs that were independently captured by the two species (Fig. 3a). While this hypothesis is in line with theory [5, 6], homomorphic and heteromorphic sex chromosomes often occur in closely related species pairs in liverworts [7, 47] (Fig. 1a), thus the possibility that the homomorphic UV chromosomes possessing a PAR and a SDR could have evolved multiple times cannot be fully ruled out. Therefore, (ii) an alternative hypothesis is that heteromorphic micro-sex chromosomes are ancestral to *M. polymorpha* and *B. pusilla* and that sex chromosomes of *B. pusilla* are derived from the fusion between an autosome and a small heteromorphic sex chromosome (Fig. 4c, FUSION).

Unfortunately, the lack of high-quality genome assemblies for species with homomorphic sex chromosomes other than *B. pusilla* (e.g. *Cavicularia*, *Treubia* and *Haplomitrium,* Fig. 1a), as well as the limited information on the phylogenetic distribution of homomorphic and heteromorphic sex chromosomes in liverworts, makes it impossible to distinguish between these two hypotheses. On the one hand, theory clearly suggests that UV sex chromosome homomorphy should be ancestral to the subclass Marchantiidae [5, 6, 43]. Nevertheless, evolution of highly heteromorphic U/V sex chromosomes, like those found in *M. polymorpha* from their homomorphic counterparts would require the loss of the PAR, and the de novo evolution of new centromeres. Studies of genetic engineering attempting to create de novo centromeres suggest that this is a complicated endeavor [48]. There is also very little experimental evidence of de novo centromere formation in natural populations of plants [49, 50]. Therefore, while the fission hypothesis is supported by theory, experimental observations suggest that chromosome fissions are extremely rarely led to viable offsprings in natural populations.

On the other hand, the origin of homomorphic sex chromosomes via sex chromosome-autosome fusions is also conceivable. Theory suggests that autosome-sex chromosome fusions may be selectively favored to establish tight linkage with sexually antagonistic genes [37, 51, 52]. Autosome-sex chromosome fusions are frequent in flowering plants and mammals [1, 53] and are hypothesized to explain some chromosome number variation in liverworts and mosses [13, 30, 47]*.* Furthermore, recent evidence from the liverwort family Ricciaceae suggests that new sex chromosomes in liverworts may rapidly evolve involving radical rearrangements of chromosomes [27, 28]. However, the mechanisms and steps by which two homologous PAR segments may fuse with the SDR of the U and V chromosomes remain unclear.

At present, our data cannot decisively distinguish between these alternatives due to the lack of information about the structure of homomorphic and heteromorphic sex chromosomes in other liverwort species. For instance, *Cavicularia densa*, a species closely related to *B. pusilla*, was suggested to have homomorphic sex chromosomes based on cytological observations [54, 55]. Information on the structure of the sex chromosomes in *Cavicularia* would help to clarify the evolutionary origins of homomorphic sex chromosomes in *B. pusilla,* as well as the mechanisms that create them. Therefore, additional genomic data from liverwort species with variable sex chromosome types and sizes (homomorphic/heteromorphic, small/large) will be needed to disentangle the ancestral state and evolutionary trajectory of sex chromosomes in the Marchantiidae subclass and in liverworts.

## Conclusions

Our study shows that the homomorphic UV sex chromosomes of the liverwort *B. pusilla* possess a well-defined PAR and SDR. This structure has never been observed in liverworts before, but we expect that it may be more widespread than previously thought. Furthermore, we hypothesize that the PAR-SDR structure and homomorphy of the UV sex chromosomes may represent an ancestral condition for the liverwort subclass Marchantiidae, and potentially for all liverworts. Consequently, heteromorphic sex chromosomes that frequently occur in liverworts may have evolved from homomorphic sex chromosomes through reduction or entire loss of the PAR. However, we acknowledge that it cannot be entirely ruled out that the homomorphic sex chromosomes of *B. pusilla* originated through fusion of an autosome and a micro-sex chromosome. Overall, we anticipate our study to stimulate further research into the evolution of sex chromosomes in liverworts, which would significantly advance our understanding of U/V sex chromosome evolution in general.

## Methods

### Plant material and genome sequencing

Male and female *Blasia pusilla* gametophytes were obtained from natural populations (Additional file 1: Table S1). A single axenic male genet from Finland (hereafter “Finnish strain”) was established by surface sterilizing ellipsoidal gemmae collected from a flask-shaped receptacle on the thallus and subsequently propagated on solid BCD [56] medium (Nuuksio National Park, Uusimaa, Finland, X. He & J. Hyvönen 4524, H4280828 (H)). For the female (“Chinese strain”), thallus tissue was collected from natural populations, carefully cleaned to remove surface contaminants, and processed immediately for DNA extraction (Additional file 1: Table S1).

Genomic DNA was extracted using the plant-EZ DNA extraction kit (Omega Bio-Tek, Norcross, Georgia). For the Finnish strain, Pacbio-SMRT sequencing was performed at the Institute of Biotechnology, University of Helsinki, generating ~ 30 Gbp of long-read data. Additional ~ 307 Gbp of short read Illumina paired-end data (NovaSeq; 2 × 150 bp) was obtained for polishing and k-mer analyses. For the Chinese strain, 2,971,204 bp ONT long-read (SQK-LSK108, PromethION Oxford Nanopore Technologies, Oxford, UK) and 773,990,635 bp Illumina short-read (NovaSeq; 2 × 150 bp) sequencing yielded approx. 115 Gbp of raw data. To improve assembly contiguity, Omni-C libraries (Dovetail Omni-C kit) were prepared for the Finnish strain, and a Hi-C library for the Chinese strain, which were both sequenced on Illumina NovaseqX platform (paired end mode, 2 × 150 bp).

### Read cleaning, genome assembly, polishing and scaffolding

For the Finnish strain, we mapped the raw reads using Blasr v5.3.5 [57] using default parameters to organelle and cyanobacterial genomes and detected substantial non-plant contamination. Therefore, we performed taxonomic binning using BlobTools v1.1.1 [58] and removed reads not classified as Viridiplantae or Streptophyte. The remaining reads were further cleaned with MEGAN [59] using DIAMOND v2.2.15 [60] in frameshift-aware mode (-F 15, range culling, –top 10), retaining only Viridiplantae-assigned reads. All cleaned reads were assembled with Flye v2.9 [61]. For the Chinese strain, the strategy of genome assembly followed [62]. Raw nanopore long reads were assembled with NextDenovo v2.4.0 (https://github.com/Nextomics/NextDenovo) with the parameter “seed_cutoff = 45”.

The cleaned and raw assembly of the Finnish strain was polished with POLCA v4.0.2 [63]. Specifically, Illumina reads were aligned to the assembly with bwa-mem v0.7.17 [64] and variants were called using FreeBayes v.1.3.2 (Variants were flagged as errors, if the alternative count > 1 and > 2 × the reference count; corrected to the highest-confidence allele) [65]. The Chinese strain`s assembly was polished using Racon v1.4.7 [66] and Pilon v1.23 [67] with nanopore long reads and short reads, respectively.

To generate a chromosome-level genome assembly for *B. pusilla*, the initial and polished assembly of the Finnish strain was scaffolded using Omni-C libraries [68]. Omni-C sequencing read pairs were aligned to the polished genome assembly using the Juicer pipeline v1.6 [69] and scaffolded with 3D-DNA v180922 [70]. Contact maps were visualized using Juicebox Assembly Tools v1.11.08, and two mis-joins were corrected manually. Chromosomes were ordered by descending scaffold length. For the Chinese strain, raw Hi-C reads were first trimmed with Trimmomatic v0.39 [71] with default parameters. Valid pairs were extracted with Juicer v1.6 [69], scaffolding was performed with the 3D-DNA pipeline v180922 [70] and mis-assemblies were curated in Juicebox Assembly Tools v1.11.08 [72]. We performed post-assembly contamination screening. Scaffolds were queried against NCBI nt (BLASTN; e-value < = 1e-5) [73]. Scaffolds with > 50% matched sites to non-Embryophyta (non-Embryophyta-hit sites/all nt-hit sites) were removed. Then, the retained scaffolds were further queried against the nr database with DIAMOND v0.9.25 [60], removing scaffolds with > 75% matched sites to non-Embryophyta. Mitochondrial and plastid sequences were removed by performing BLASTN search against organellar sequences from three model bryophytes (i.e., *Marchantia polymorpha*, *Physcomitrium patens,* and *Anthoceros angustus*). The filtered Chinese strain`s genome assembly was further scaffolded against the nine chromosomes of the Finnish assembly using RagTag v1.2.0 [74] with default options.

### Identification of telomeric repeats

To identify telomeric repeats, Tidk v0.2.65 was used [75]. First, the Tidk ‘explore’ was ran on the female assembly to identify 5—12 bp long putatively telomeric monomers. Twelve monomers were identified with Tidk ‘search’, and the results were plotted with Tidk ‘plot’. Seven monomers (AGGGTTTT, AGGGGTTT, AGGGTTT, AGGTTTAGGTTT, AGGTTT, AGGGTT, GGGCCT) showed enrichment toward chromosome ends and were therefore used to re-run Tidk ‘search’ with a 250 kb window (-w 250,000). Combined monomer distributions were plotted using ggplot2 v3.5.1 in R v4.3.3 [76].

### Transposable element and repeat annotation

Repetitive elements in the *B. pusilla* genome were identified using both de novo and homology-based approaches. A masking library for gene predictions was generated with RepeatModeler v2.0.2 [77] using default parameters and merged with the RepBase [78] plant library (https://www.girinst.org/). RepeatMasker v4.1.2 [79] was used to soft-mask the genome. For the comprehensive TE annotation (both female and male strains), we used EDTA v2.1.3 (https://github.com/oushujun/EDTA) [80], which combines structural and homology-based TE discovery. Additional RepeatModeler consensus sequences were merged into a non-redundant TE library and classified using TEsorter v1.3.0 [81]. The final annotation was performed with RepeatMasker v4.11 [79] with default parameters. For cross-species comparisons, the *M. polymorpha* reference genome (version MpTak v6.1r2 retrieved from https://marchantia.info/) was re-annotated using the same EDTA workflow. TE landscapes were obtained from RepeatMasker outputs with parseRM_GetLandscape.pl (https://github.com/4ureliek/Parsing-RepeatMasker-Outputs).

### Gene prediction and UTR refinement

To aid gene prediction, RNA was extracted from axenically cultured gametophytes of the *B. pusilla* female plant (Potsdam strain), provided by Prof. Elke Dittman (University of Potsdam). This RNA-seq data contained 42 samples collected at different time points under control and nitrogen-starved conditions [82]. Total RNA was extracted using the Spectrum Plant Total RNA Kit (Sigma-Aldrich) and strand-specific RNA-seq libraries were prepared with the Truseq stranded mRNA Library Prep kit (Illumina), and sequenced on the Illumina Novaseq 6000 (2 × 150 bp) by Novogene UK. Raw reads were quality checked using FastQC v0.12.1 [83] and trimmed using default options with Trimmomatic v0.39 [71].

Gene annotation was performed using Braker2 v2.1.6 [84] in combination with TSEBRA v1.1.2.3 [85]. RNA-seq reads were mapped to the soft-masked genome assembly using HISAT2 v2.2.1 [86], and an initial Braker2 run was performed using RNA-seq evidence to train GeneMark-ET [87] and Augustus v3.5.0 [88]. A second braker2 run incorporated protein evidence from OrthoDB v12.1 [89] and six additional bryophyte proteomes (*Anthoceros agrestis*, *A. punctatus*, *Marchantia polymorpha*, *M. paleacea*, *Phsycomitrium patens* and *B. pusilla* de novo assembly [82]. Following the best practices recommendations, RNA-only and protein-only gene predictions were merged using TSEBRA v1.1.2.3 [85], lowering the “intron-support” threshold to 0.2 to retain falsely removed gene models. The merged GFF was exported using Gffread v0.12.7 [90] and untranslated regions (UTRs) were added to the gene annotation with Gemoma v1.9 AnnotationFinalizer (https://www.jstacs.de/index.php/GeMoMa) [91]. Prior to this UTR refinement step, intron/coverage evidence was computed from alignments using GeMoMa ERE. AnnotationEvidence assigned transcript-level attributes (tie, tpc, amino-acid sequences, start/stop codons). IDs were standardized with Genometools v1.6.5 (gt rename) [92]. Final CDS, transcript and protein fasta files were extracted with Gffread v0.12.7 [90]. Final gene models were filtered using Transdecoder v5.7.1 (https://github.com/TransDecoder/TransDecoder), retaining only complete ORF (canonical start/stop codons, no in-frame stop codons). A small number of missing but well-supported sex-linked genes were manually added to the final annotation.

Gene prediction of the Chinese strain was also performed with Braker v2.1.5 [84]. After repeat masking, Braker integrated the transcriptome-based, homology-based, and ab initio evidence. Protein sequences of six green plants (i.e., *Arabidopsis thaliana*, *Azolla filiculoides*, *Marchantia polymorpha*, *Physcomitrium patens*, *Salvinia cucullata*, and *Selaginella moellendorffii*) retrieved from the Phytozome v13 database (https://phytozome-next.jgi.doe.gov/) were used as the homology-based evidence.

### Statistical comparison of genomic features of the SDR, PAR, and the autosomes

We extracted genomic features (see in the Results section) from the gff file of the *B. pusilla* male accession (Finnish strain), and compared their abundance among the PAR (Chr_09: 0—21.41 Mbp), SDR (Chr_09: 21.41 Mbp-end of the chromosome) and the autosomes. For the statistical comparisons, we used pairwise Mann–Whitney U tests. To investigate whether the PAR is enriched for orphan genes, we performed blast searches of (BLASTP, e-value < = 10^–4^) PAR and autosomal genes against the proteome containing all liverwort proteins from the 1Kp transcriptome and those from bryogenome.org [62]. We compared the proportion of genes without significant hit against the proteome (e-value < = 10^–4^) on the PAR with that of the autosomes using a chi-square test. In addition to using all autosomes, we also plotted the results for Chr_05 of *B. pusilla*, which is similar in size as well as gene number to Chr_09.

### Whole genome alignments and collinearity/synteny

Genome comparisons between *B. pusilla* accessions (Finnish vs. Chinese) and between *B. pusilla* and *M. polymorpha* were carried out by creating dot plots in D-GENIES v1.3.0 [93] from Minimap2 v2.28 pairwise genome alignments [94]. We applied a minimum identity threshold of 90% between the two *B. pusilla* strains and 70% for cross-species comparisons (*B. pusilla* vs *M. polymorpha*). Structural variation between *B. pusilla* and *M. polymorpha* assemblies was further called from the NUCmer module from MUMer v4.0.0 [95] alignments and summarized using Assemblytics v1.2.1 [96].

Collinearity was assessed with MCScanX toolkit [97] (distance = 80, minimum gene number = 6, --full). Ka/Ks for syntenic gene pairs were computed using MCScan utilities. Collinearity within genomes as well as genome-wide feature distributions were visualized with Circos v0.52 [98], SynViso (https://synvisio.github.io/) and the Python version of MCScan (https://github.com/tanghaibao/jcvi) [99].

### Phylogenetic analysis of gametologs

Orthogroups were inferred using OrthoFinder v2.5.5 [100] using proteomes from 30 liverworts species (Additional file 1: Table S6). Gene trees containing *M. polymorpha* gametologs [15] were extracted for phylogenetic analysis (Additional file 2: Fig. S9). Protein alignments (MAFFT v7) [101] were used to infer maximum-likelihood trees using iqTree v2 [102] (http://iqtree.cibiv.univie.ac.at/) and visualized with the R package ggtree [103].

### Functional annotation

Functional annotation of predicted genes was performed using two complementary approaches. Gene Ontology (GO) terms and functional domains were assigned using eggNOG-mapper v2.1.12 [104], and additional functional classification was obtained using Trapid v2.0 [105]. Annotated gene models are provided in Additional file 1: Table S9.

## Supplementary Information

Additional file 1: Table S1. Information on the *Blasia pusilla* accessions sequenced. Table S2. Raw and filtered read data used to assemble the *B. pusilla* genomes. Table S3. Genome size estimates and assembly statistics of the *B. pusilla* assemblies used. Table S4. Genome annotation statistics and BUSCO assessment of the two *B. pusilla* genomes. Table S5. Repeat annotation of the two *B. pusilla* strains and that of *Marchantia polymorpha*. Table S6. Proteomes used for comparative orthogroup analysis of *B. pusilla* and other liverworts species. Table S7. Homologs of *M. polymorpha* U- and V-linked genes in the *B. pusilla* male and female genomes. Table S8. *M. polymorpha* homologs of *B. pusilla* SDR genes not homolgous to *M. polymorpha* gametologs and UV-specific genes. Table S9. EggNOG-mapper annotatiion of *B. pusilla *genes. Table S10. Pairwise estimates of Ka, Ks, and Ka/Ks for male and female gene pairs in *B. pusilla*.

Additional file 2: Fig. S1. Genome size estimates of the *B. pusilla *genomes. Fig. S2. Hi-C contact maps for the *B. pusilla* assemblies. Fig. S3. Comparison of structural variation between the two *B. pusilla* genomes. Fig. S4. Distribution of telomeric repeats in the two *B. pusilla* genomes. Fig. S5. Syntenic depth analysis of the *B. pusilla*, Finland, male and the *Marchantia polymorpha *genomes. Fig. S6. Structural characteristics of the PAR compared with autosomes, Bp_5, and the SDR in the *Blasia pusilla* male accession. Fig. S7. The PAR of *B. pusilla* is enriched for orphan genes. Fig. S8. Abundance of TE classes on the chromosomes of *B. pusilla*, Finland, male strain. Fig. S9. Phylogenetic trees of the 20 *M. polymorpha* gametolog pairs, their homologs in *B. pusilla*, and other liverworts. Fig. S10. Repeat landscapes for the *B. pusilla* genome.

## Data Availability

Raw DNA sequencing data used in this publication was submitted to NCBI Short Read Archive (SRA) under the BioProject ID: PRJNA1302427 [106]. Raw RNA data used in this publication was submitted to SRA under the BioProject ID: PRJNA1099914 [107]. The HiC sequencing data for both *Blasia pusilla* male and female strains were submitted to NCBI SRA under BioProject ID: PRJNA1302427 [106]. The genome assemblies as well as gametolog trees are available on Figshare (10.6084/m9.figshare.32076294) [108] and (10.6084/m9.figshare.27015028) [109], respectively. The *B. pusilla* female Dong, *B. pusilla* female Potsdam, and *B. pusilla* male Helsinki, transcriptome assemblies are also available on Figshare (10.6084/m9.figshare.27015028) [109]. The previously published transcriptomes of *Radula lindenbergiana, Treubia lacunosa, Sphaerocarpos texanus, Ptilidium pulcherrimum, Conocephalum conicum, Scapania nemorosa, Pellia neesiana, Porella navicularis, Schistochila_sp, Marchantia paleacea, Marchantia polymorpha, Metzgeria carssipilis, Barbilophozia barbata, Pellia epiphylla, Calypogeia fissa, Monoclea gottschei, Marchanthia emarginata, Frullania-sp, Porella pinnata, Ricciocarpos natans, Bazzania trilobata, Odontoschisma prostratum, Pallavicinia lyellii* were retrieved from the 1Kp database (https://ftp.cngb.org/pub/SciRAID/onekp/assemblies/) [110] whereas the male and female *Lunularia cruciata* assemblies, gene sets and alignments were downloaded from genomeevolution.org and the original publication (https://genomevolution.org/coge/SearchResults.pl?s=64629&p=genome, https://academic.oup.com/gbe/article/15/3/evad014/7023155supplementary data) [111, 112]. The *Marchantia polymorpha* genome data was downloaded from marchantia.info (https://marchantia.info/download/MpTak_v6.1r2/) [113]. Proteomes of *Anthoceros agrestis* (https://www.hornworts.uzh.ch/en/download.html) [114], *A. punctatus* (https://www.hornworts.uzh.ch/en/download.html) [114], *Marchantia polymorpha* v7.1 (https://marchantia.info/download/) [115], *M. paleacea* (https://www.ncbi.nlm.nih.gov/datasets/genome/GCA_014180765.2/) [116], *Physcomitrium patens* (http://phytozome-next.jgi.doe.gov/info/Ppatens_v7_1) [117], *Arabidopsis thaliana* (https://phytozome-next.jgi.doe.gov/info/Athaliana_Araport11) [118], *Azolla filiculoides* (https://fernbase.org/ftp/) [119], *Salvinia cucullata* (https://fernbase.org/ftp/) [119], and *Selaginella moellendorffii* (https://phytozome-next.jgi.doe.gov/info/Smoellendorffii_v1_0) [120] were retrieved from public databases. Further liverwort proteomes were downloaded from bryogenome (http://www.bryogenomes.org/) [121]. The chloroplast and mitochondrial genomes of *Marchantia polymorpha* (https://marchantia.info/download/organellar/) [122], *Physcomitrium patens* (https://www.ncbi.nlm.nih.gov/nuccore/AP005672, https://www.ncbi.nlm.nih.gov/nuccore/NC_007945) [123, 124], and *Anthoceros angustus* (https://www.ncbi.nlm.nih.gov/nuccore/AB086179, https://www.ncbi.nlm.nih.gov/nuccore/MG029262) [125, 126] were obtained from public databases. Bioinformatic analyses and scripts are available in GitHub (https://github.com/joleneyue/Genome_Blasiapusilla/tree/main) [127] and in Figshare (10.6084/m9.figshare.32135128) [128] under the MIT license.
