# Supplementary material for: Genome assemblies of the liverwort Blasia pusilla uncover a well-defined pseudoautosomal region on homomorphic UV sex chromosomes
Source: Genome Biol. 2026 May 18;27:166. doi: 10.1186/s13059-026-04101-2 (PMC13182070; doi:10.1186/s13059-026-04101-2)
Supplement: Supplementary file 2 — Additional file 2: Fig. S1. Genome size estimates of the B. pusilla genomes. Fig. S2. Hi-C contact maps for the B. pusilla assemblies. Fig. S3. Comparison of structural variation between the two B. pusilla genomes. Fig. S4. Distribution of telomeric repeats in the two B. pusilla genomes. Fig. S5. Syntenic depth analysis of the B. pusilla, Finland, male and the Marchantia polymorpha genomes. Fig. S6. Structural characteristics of the PAR compared with autosomes, Bp_5, and the SDR in the Blasia pusilla male accession. Fig. S7. The PAR of B. pusilla is enriched for orphan genes. Fig. S8. Abundance of TE classes on the chromosomes of B. pusilla, Finland, male strain. Fig. S9. Phylogenetic trees of the 20 M. polymorpha gametolog pairs, their homologs in B. pusilla, and other liverworts. Fig. S10. Repeat landscapes for the B. pusilla genome. [file 13059_2026_4101_MOESM2_ESM.pdf]

Supplementary Figures

Article title: Genome assemblies of the liverwort *Blasia pusilla* uncover a well-defined pseudoautosomal region on homomorphic UV sex chromosomes

Authors: *Yuling Yue[1,2], Gaurav Sablok[3], Xiaolan He[3], Jaakko Hyvönen[3]*, *Shanshan Dong[4], Giacomo Potente[1,2], Yang Liu[4], Péter Szövényi[1,2]*

1 Department of Systematic and Evolutionary Botany, University of Zurich, Switzerland, 8008, Zollikerstrasse 107

2 Zurich-Basel Plant Science Center, Zurich, Switzerland

3 Botany and Mycology Unit, Finnish Museum of Natural History, PO Box 7, FIN-00014 University of Helsinki, Finland

4 Key Laboratory of Southern Subtropical Plant Diversity, Fairy Lake Botanical Garden & Chinese Academy of Sciences, Shenzhen, China.

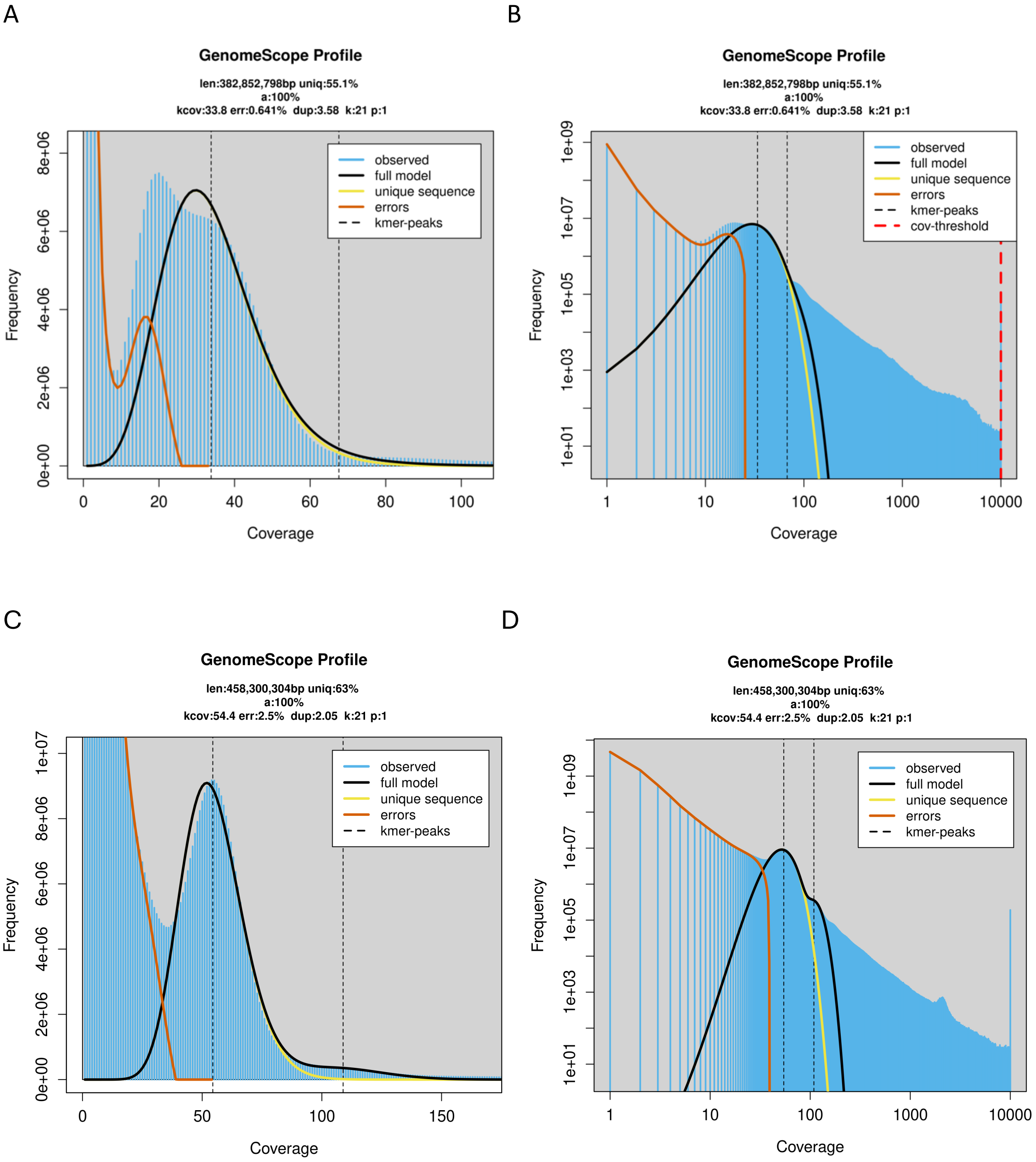

Fig. S1. Genome size estimates of the *Blasia pusilla* (Finland male and Chinese female strain) genomes. Size estimates were obtained using GenomeScope 2.0 with k-mer=21. (A) and (B): *B. pusilla* Finland male genome. (C) and (D): *B. pusilla* Chinese female genome.

A

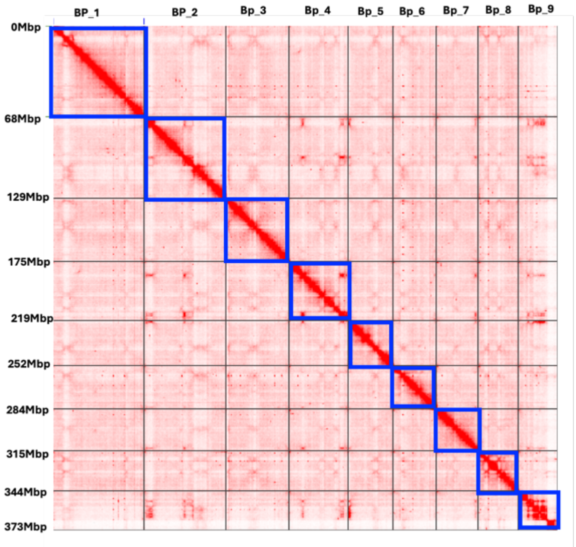

B

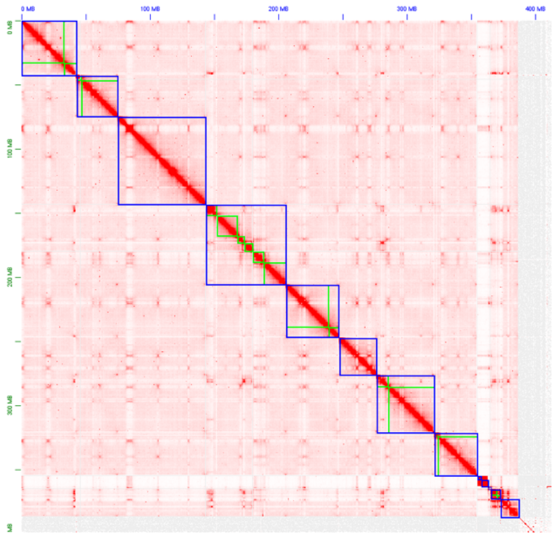

Fig. S2. Hi-C contact maps for the *B. pusilla* assemblies (Finland male and Chinese female strain). (A) *B. pusilla* male genome with the nine expected chromosomes. (B) Hi-C scaffolding of the *B. pusilla* Chinese female genome is shown prior to scaffolding on the male assembly. Hi-C scaffolding of the *B. pusilla* Chinese female genome led to a less contiguous assembly containing 14 scaffolds.

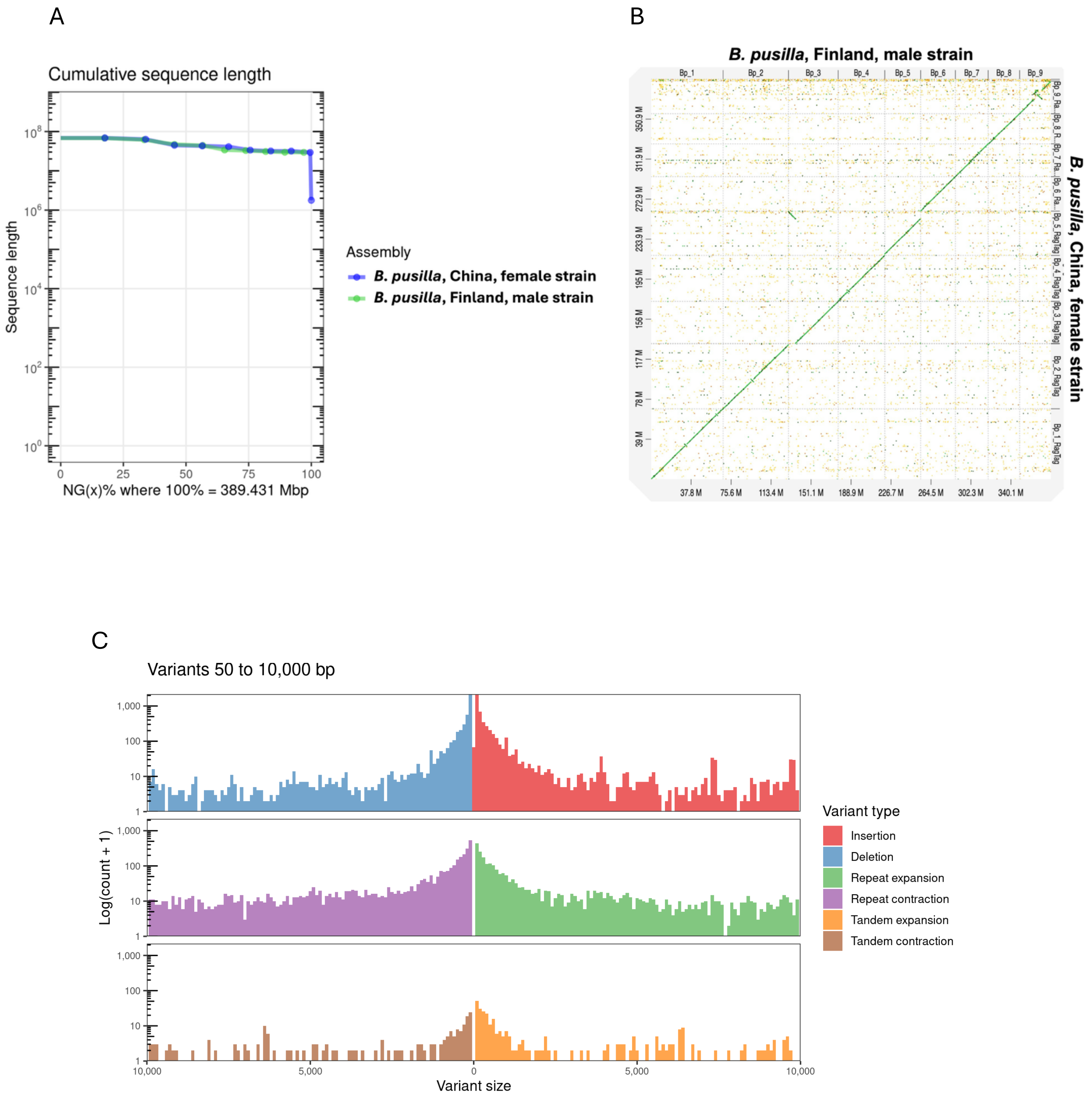

Fig. S3. Comparison of structural variation between the two *B. pusilla* genomes (Finland male and China female strains). (A) Comparison of assembly contiguity of the male and female *B. pusilla* assemblies (Assemblytics). (B) Dot plot of the male and female *B. pusilla* assemblies (D-GENIES, only matches with a similarity greater than 70% are shown). (C) Length and frequency distribution of structural variants between the male and the female *B. pusilla* assemblies. Variants were classified and plotted using Assemblytics.

A

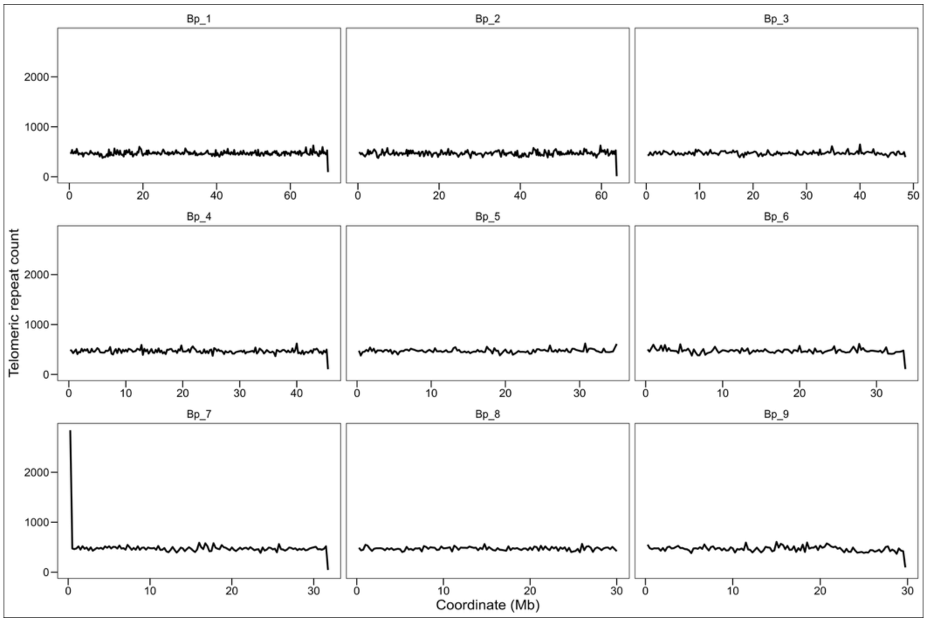

B

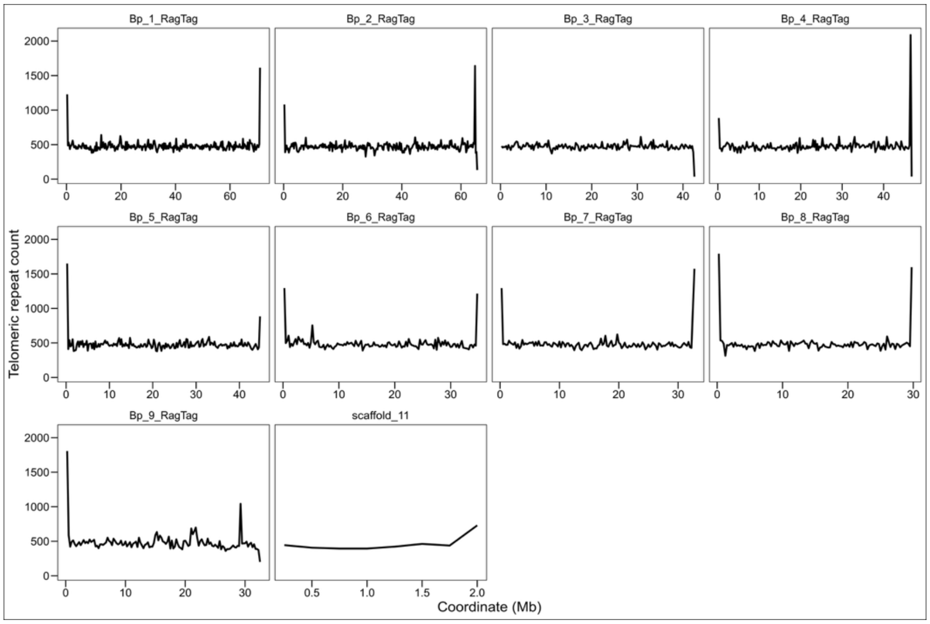

Fig. S4. Distribution of telomeric repeats in the two *B. pusilla* genomes. (A) Finland, male; (B) China, female. Telomeric repeat (“TTTAGGG”) count is shown for each chromosome in 250 kb windows. Telomers are better assembled in the female genome.

A B

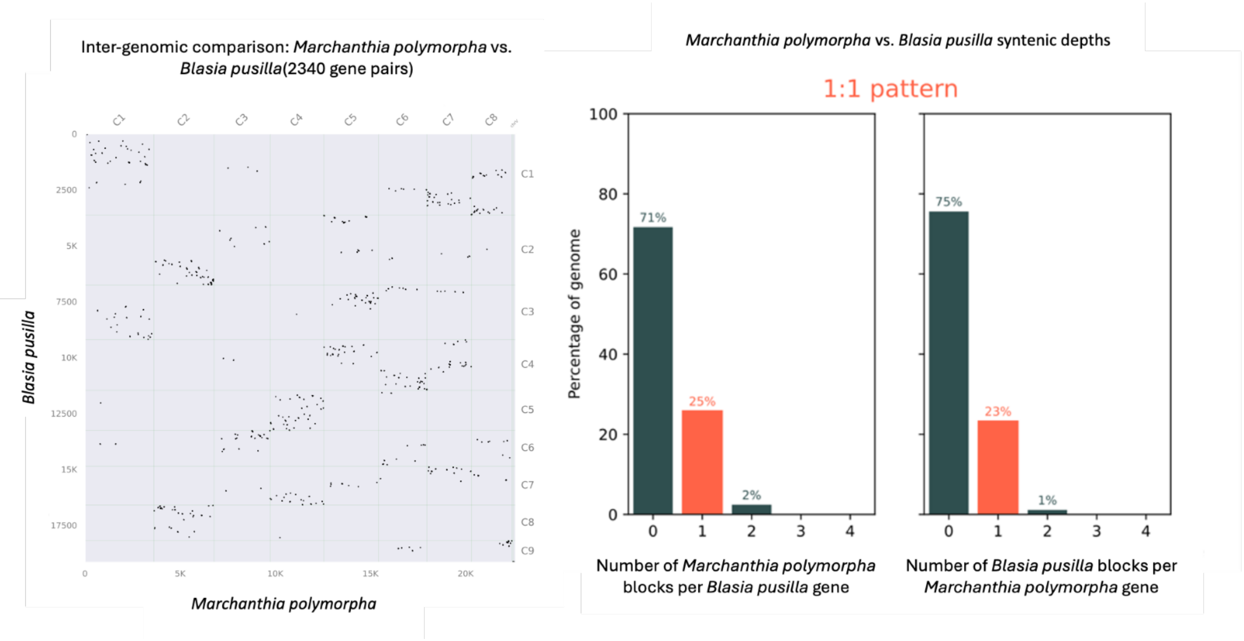

Fig. S5. Syntenic depth analysis of the *B. pusilla*, Finland, male and the *Marchantia polymorpha* (MpTakv6.1r2) genomes. Dot plot between the *M. polymorpha* and *B. pusilla* genomes. (A) Distribution of the number of *M. polymorpha* syntenic blocks per *B. pusilla* gene and the number of *B. pusilla* syntenic blocks per *M. polymorpha* gene. (B) Percentages indicate the proportion of the genome corresponding to each category.

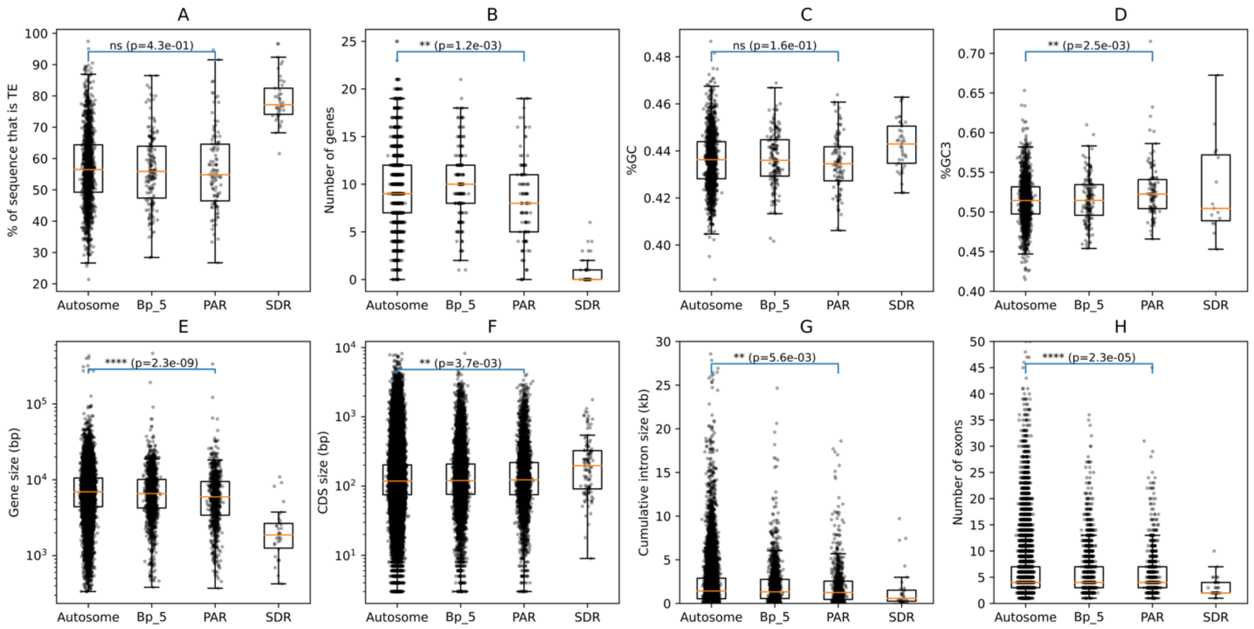

Fig. S6. Structural characteristics of the PAR compared with autosomes, Bp_5, and the SDR in the *Blasia pusilla* male (Chinese) accession. For window-based analyses (A–D), values were calculated using 200kb non overlapping genomic windows. The PAR was defined as positions 0–21.41 Mbp on Bp_9 whereas the remaining portion of the chromosome as the SDR. (A) Percentage of sequence occupied by TEs. (B) Gene density (gene/200 Kb) per genomic window. (C) GC content (%GC) per window. (D) GC content at third codon positions (%GC3) per coding sequence overlapping each window. (E) Gene size per gene. (F) Coding sequence (CDS) size per gene. (G) Total intron length per gene. (H) Number of exons per gene. Statistical differences between the PAR and autosomes were assessed using Mann–Whitney U tests; outcomes of the tests are indicated on each panel. Red line: median, boxes: interquartile range (IQR), whiskers: 1.5 x IQR.

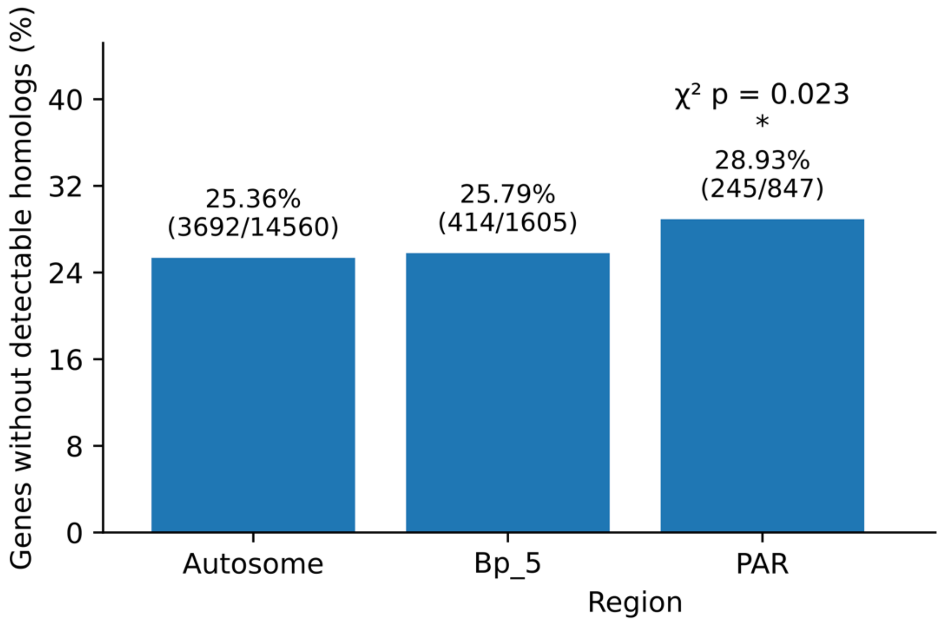

Fig. S7. The PAR of *B. pusilla* is enriched for orphan genes. We used the *B. pusilla* male (Chinese) accession and defined the PAR in a similar way as in Supplementary Figure 6. Difference in the proportion of orphan genes (no BLASTP hit against a large data set of liverwort proteomes with an e-value threshold of 10^{-4}) between the PAR and the autosomes was tested using a chi-square test. P-values are shown above the PAR bars ($p < 0.05$).

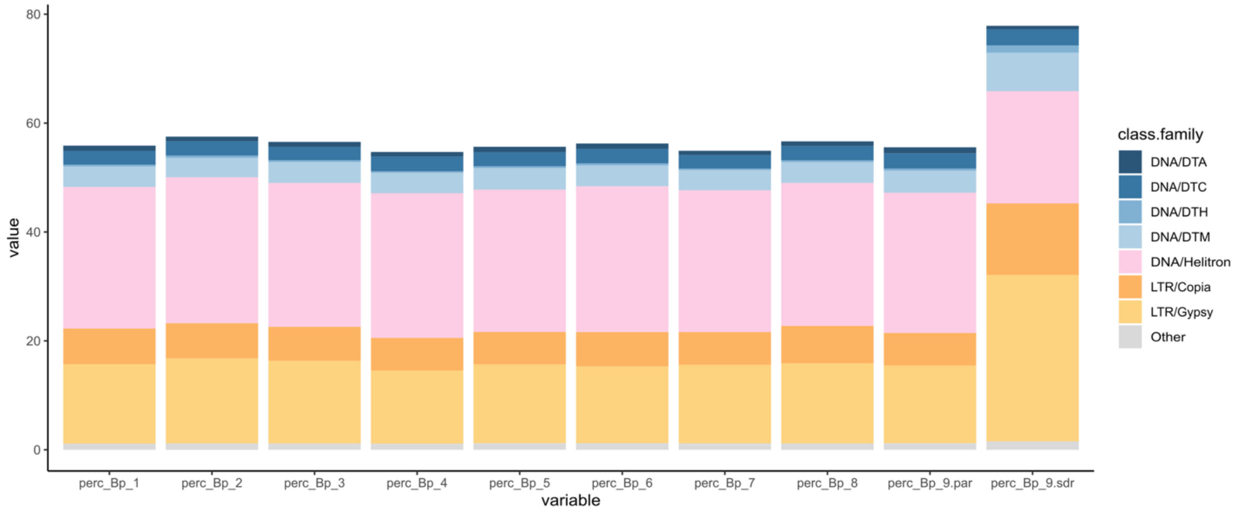

Fig. S8. Abundance of TE classes on the chromosomes of *B. pusilla*, Finland, male strain. TE classes and families are shown in different colors. The pseudoautosomal (PAR, 0-21.4 Mbp) and sex determining region (SDR, 21.4-30 Mbp) of chromosome nine are shown separately.

MpU00010

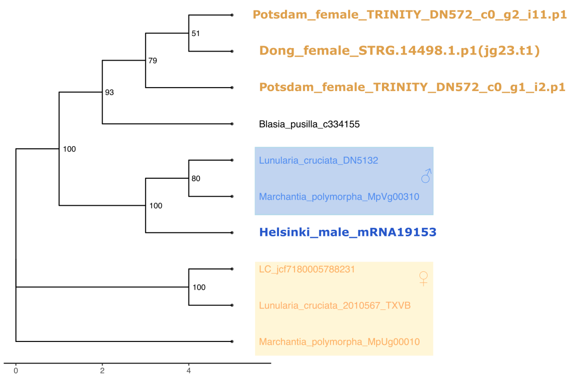

MpU00020

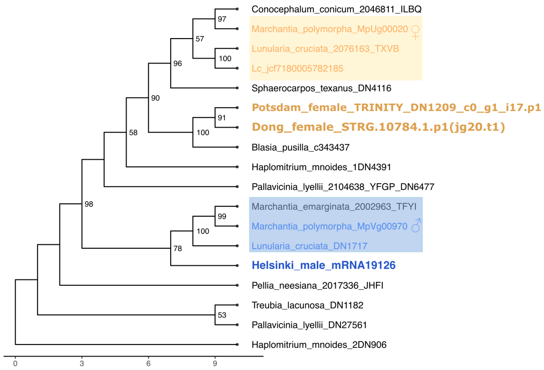

MpU00030

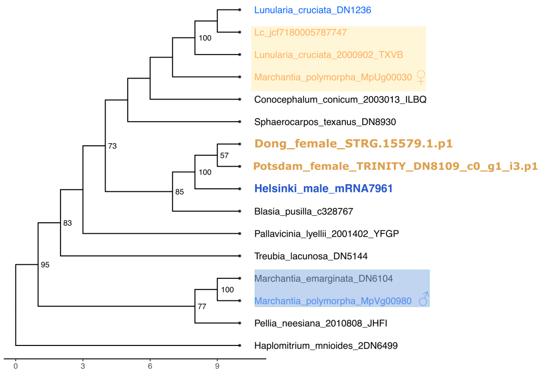

MpU00050

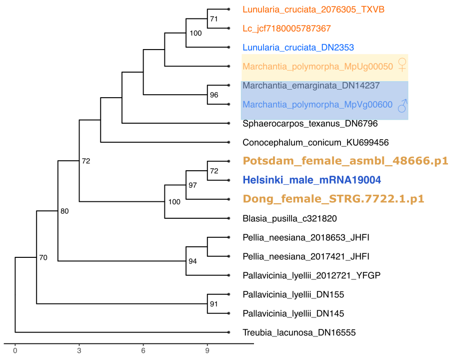

MpU00060

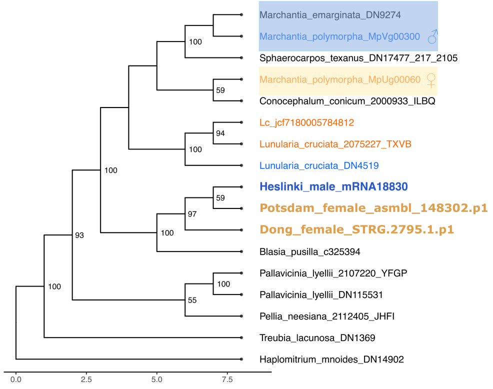

MpU00090_420

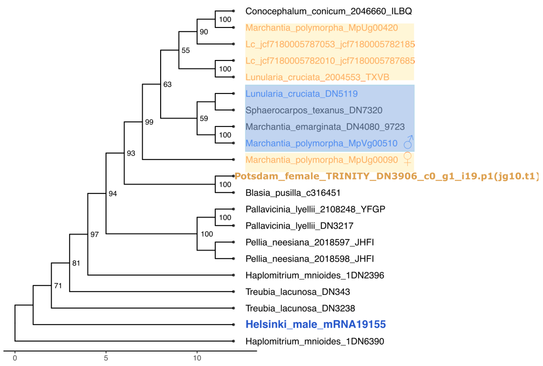

MpU00100

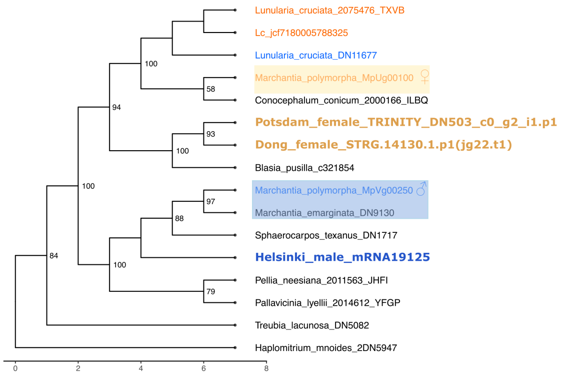

MpU00110

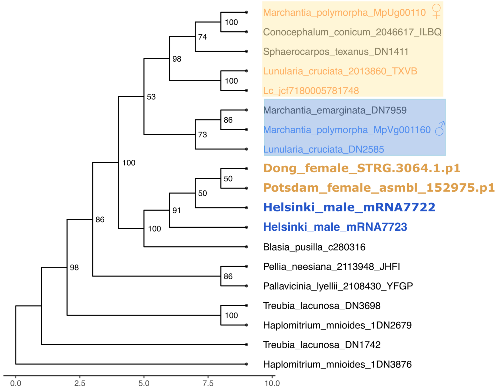

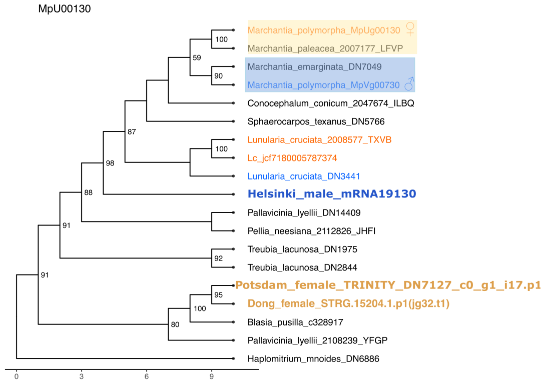
MpU00130
Marchantia_polymorpha_MpUg00130 ♀
Marchantia_paleacea_2007177_LFVP
Marchantia_emarginata_DN7049
Marchantia_polymorpha_MpVg00730 ♂
Conocephalum_conicum_2047674_ILBQ
Sphaerocarpos_texanus_DN5766
Lunularia_cruciata_2008577_TXVB
Lc_jcf7180005787374
Lunularia_cruciata_DN3441
Helsinki_male_mRNA19130
Pallavicinia_lyellii_DN14409
Pellia_neesiana_2112826_JHFI
Treubia_lacunosa_DN1975
Treubia_lacunosa_DN2844
Potsdam_female_TRINITY_DN7127_c0_g1_i17.p1
Dong_female_STRG.15204.1.p1(jg32.t1)
Blasia_pusilla_c328917
Pallavicinia_lyellii_2108239_YFGP
Haplomitrium_mnoides_DN6886

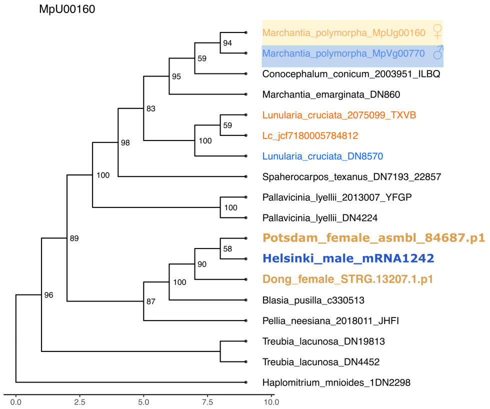
MpU00160
Marchantia_polymorpha_MpUg00160 ♀
Marchantia_polymorpha_MpVg00770 ♂
Conocephalum_conicum_2003951_ILBQ
Marchantia_emarginata_DN860
Lunularia_cruciata_2075099_TXVB
Lc_jcf7180005784812
Lunularia_cruciata_DN8570
Spaherocarpos_texanus_DN7193_22857
Pallavicinia_lyellii_2013007_YFGP
Pallavicinia_lyellii_DN4224
Potsdam_female_asmbl_84687.p1
Helsinki_male_mRNA1242
Dong_female_STRG.13207.1.p1
Blasia_pusilla_c330513
Pellia_neesiana_2018011_JHFI
Treubia_lacunosa_DN19813
Treubia_lacunosa_DN4452
Haplomitrium_mnioides_1DN2298

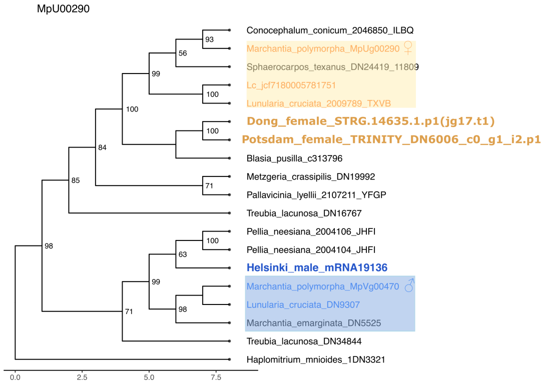
MpU00290
Conocephalum_conicum_2046850_ILBQ
Marchantia_polymorpha_MpUg00290 ♀
Sphaerocarpos_texanus_DN24419_11809
Lc_jcf7180005781751
Lunularia_cruciata_2009789_TXVB
Dong_female_STRG.14635.1.p1(jg17.t1)
Potsdam_female_TRINITY_DN6006_c0_g1_i2.p1
Blasia_pusilla_c313796
Metzgeria_crassipilis_DN19992
Pallavicinia_lyellii_2107211_YFGP
Treubia_lacunosa_DN16767
Pellia_neesiana_2004106_JHFI
Pellia_neesiana_2004104_JHFI
Helsinki_male_mRNA19136
Marchantia_polymorpha_MpVg00470 ♂
Lunularia_cruciata_DN9307
Marchantia_emarginata_DN5525
Treubia_lacunosa_DN34844
Haplomitrium_mnioides_1DN3321

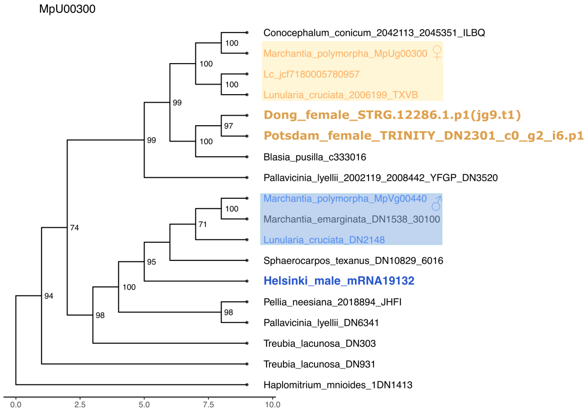
MpU00300
Conocephalum_conicum_2042113_2045351_ILBQ
Marchantia_polymorpha_MpUg00300 ♀
Lc_jcf7180005780957
Lunularia_cruciata_2006199_TXVB
Dong_female_STRG.12286.1.p1(jg9.t1)
Potsdam_female_TRINITY_DN2301_c0_g2_i6.p1
Blasia_pusilla_c333016
Pallavicinia_lyellii_2002119_2008442_YFGP_DN3520
Marchantia_polymorpha_MpVg00440 ♂
Marchantia_emarginata_DN1538_30100
Lunularia_cruciata_DN2148
Sphaerocarpos_texanus_DN10829_6016
Helsinki_male_mRNA19132
Pellia_neesiana_2018894_JHFI
Pallavicinia_lyellii_DN6341
Treubia_lacunosa_DN303
Treubia_lacunosa_DN931
Haplomitrium_mnioides_1DN1413

MpU00350

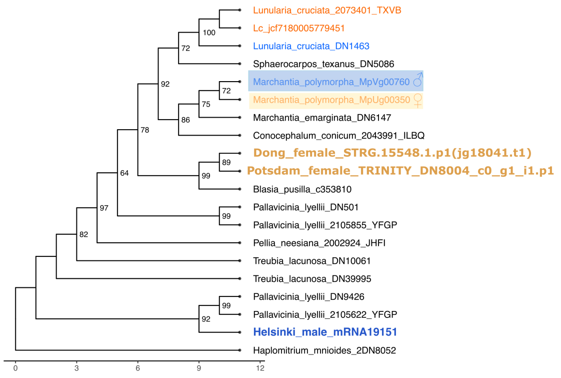

MpU00360

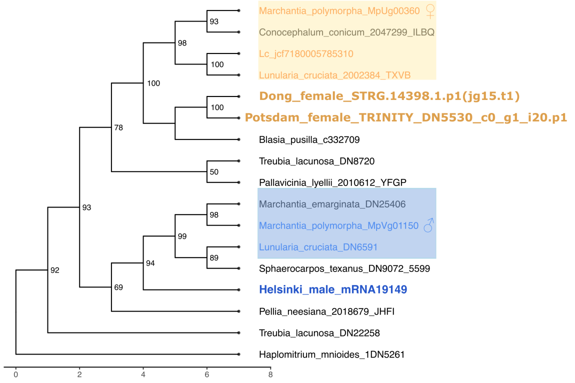

MpU00370

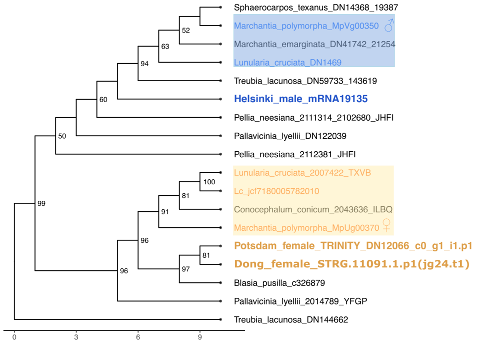

MpU00410

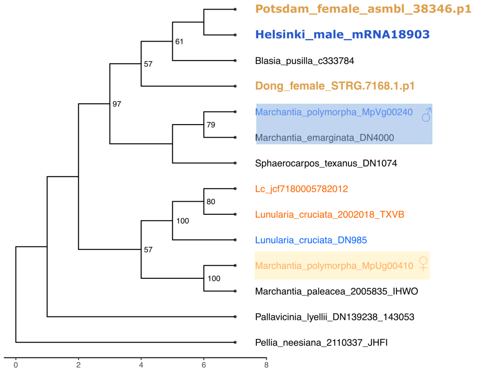

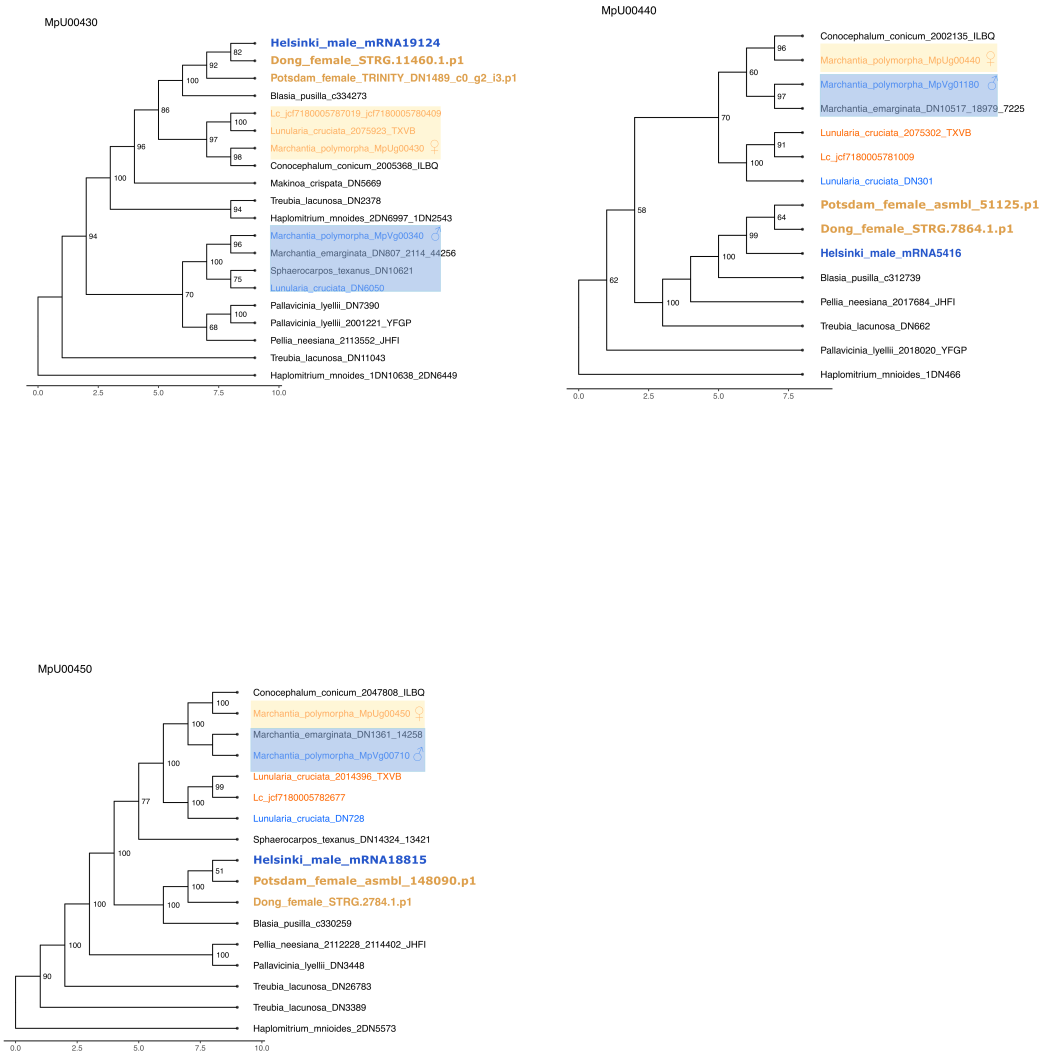

Fig. S9. Phylogenetic trees of the 20 *M. polymorpha* gametolog pairs, their homologs in *B. pusilla* and other liverworts. Clades containing the *M. polymorpha* U or the V gametolog are highlighted in yellow ♀, and blue ♂, respectively. Gene ids of female and male *L. cruciata* and *M. polymorpha* are shown in yellow (female) or blue (male), respectively. *B. pusilla* sequences from 2 female transcriptomes and the male genome are enlarged, in bold font and colored in yellow (female) or blue (male), respectively. Gene ids of the female assembly are also indicated in brackets. Bootstrap values from 1000 fast replicates are shown at each node. The x axis indicates branch length.

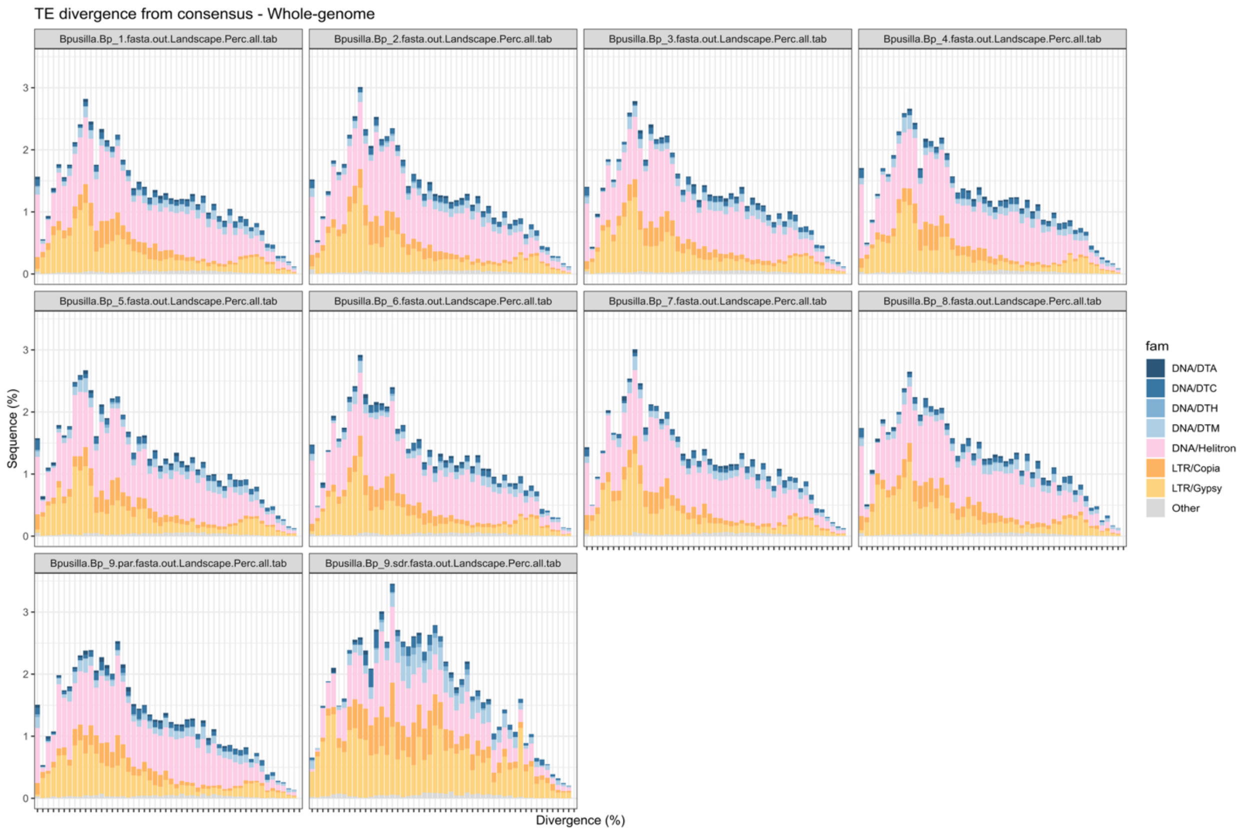

Fig. S10. Repeat landscapes for the *B. pusilla* genome. Repeat landscape for each *B. pusilla* autosome (Bp_01-08) is shown separately. For the sex chromosome (Bp_09) repeat landscapes of the pseudoautosomal (PAR) and the sex determining region (SDR) are separately depicted. Plots show the abundance of TEs (Y-axis: sequence (%)) in each sequence divergence class (X-axis: Divergence (%)) from the consensus. Major TE families are shown with different colors.
